# Evaluation of Inflammation by Cytokine Production Following Combined Exposure to Ultraviolet and Radiofrequency Radiation of Mobile Phones on 3D Reconstructed Human Skin In Vitro

**DOI:** 10.3390/ijerph17124401

**Published:** 2020-06-19

**Authors:** Zsófia Szilágyi, Zsuzsanna Németh, József Bakos, Péter Pál Necz, Anna Sáfár, Györgyi Kubinyi, Brahim Selmaoui, György Thuróczy

**Affiliations:** 1 Department of Non-ionizing radiation, National Public Health Center, H-1221 Budapest, Hungary; szilagyi.zsofia@osski.hu (Z.S.); nemeth.zsuzsanna@osski.hu (Z.N.); necz.peter@osski.hu (P.P.N.); safar.anna@osski.hu (A.S.); kaszas@hp.osski.hu (G.K.); thuroczy.gyorgy@osski.hu (G.T.); 2 Department of Experimental Toxicology, National Institute of Industrial Environment and Risks (INERIS), 60550 Verneuilen Halate, France; brahim.selmaoui@ineris.fr; 3 PériTox Laboratory, UMR-I 01 INERIS, Picardie Jules Verne University, 80025 Amiens, France

**Keywords:** radiofrequency, ultraviolet radiation, skin, combined exposure, adaptive response, mobile phone, 3G

## Abstract

The absorption of exposure to radiofrequency (RF) emitted by wireless devices leads to a high specific absorption rate in the skin. Ultraviolet (UV) radiation can induce several damages to the skin. The aim of this study was to examine whether combined, consecutive exposure to solar UV radiation and 1950 MHz RF exposure of third generation (3G) mobile system have any effect on inflammation processes in the skin. Under in vitro experiments, the inflammation process was examined by cytokines (IL-1α, IL-6, and IL-8) and MMP-1 enzyme secretion on 3D full thickness human skin model. The RF exposure was applied before or after UV irradiation, in order to study either the possible cooperative or protective effects of exposure to RF and UV. We did not find changes in cytokines due to exposure to RF alone. The RF exposure did not enhance the effects of UV radiation. There was a statistically not-significant decrease in cytokines when the skin tissues were pre-exposed to RF before being exposed to 4 standard erythemal dose (SED) UV compared to UV exposure alone. We found that RF exposure reduced the previously UV-treated MMP-1 enzyme concentration. This study might support the evaluation of the effects on the skin exposed to microwave radiation of 5G mobile technology.

## 1. Introduction

### 1.1. RF Exposure of Mobile Wireless Technology

With the rapid development of mobile communications, globally there were almost 8 billion mobile phone subscriptions at the end of 2019, as estimated by the Ericsson Mobility Report [1]. The exposure to radio frequency (RF) electromagnetic fields (EMF) emitted by mobile telephony has become a major public health concern. Therefore, it is necessary to investigate and understand its potential health impact. Most epidemiological investigations have looked for an association between brain tumors and mobile phone use, and the overall evidence indicates a slightly increased risk, despite several methodological concerns that appeared. The working group of the International Agency for Research on Cancer (IARC) classified RF–EMF as “possibly carcinogenic to humans”, (Group 2B) in view of the limited evidence in humans and in experimental animals [2]. The exposure of the human skin to radiofrequency emitted by wireless devices is considerable, since the exposures to higher RF frequency lead to higher specific absorption rates (SAR) in the skin. On the other hand, incidences of skin cancer is increasing worldwide, especially within older people. Solar ultraviolet radiation has adverse effects, including sunburn, development of immunosuppression, and skin cancers. Ultraviolet (UV) radiation directly and indirectly induces DNA lesions, which cause mutations and trigger inflammation and immunosuppression that mediate tumor growth. The IARC therefore classified UV and solar radiation as “carcinogenic to humans” (Group 1) [3].

The new generations’ wireless technologies (3G, 4G, WiFi) use higher frequencies than GSM 900 MHz systems. The fifth generation (5G) mobile technology use frequencies beyond 6 GHz, up to millimeter waves (MMW). As frequency increases, the penetration depth into the human tissue decreases, which means that most of the RF power (SAR) is absorbed in the skin of the body [4]. Skin has high water content, so the RF or microwave absorption in the skin is higher than the other organs with lower water content [5,6]. Another matter of consideration is that at a higher frequency, the absorption is only on the skin, other organs are not involved because of the small penetration depth. Therefore, the skin exposure might have an important role in the possible health effects of RF radiation and will take an important role in the introduction of 5G mobile services [7]. The analysis of superficial tissues from modeled commercial sources (i.e., mobile phone) that operated in the 900 and 1800 MHz bands demonstrated that exposure of skin tissues might well exceed 40 W/kg at the cell level [8]. In addition, the body-worn new emerging wireless devices also expose the skin to radiation [9]. The WHO Fact sheet No. 193. (reviewed in October, 2014) says: “At the frequencies used by mobile phones, most of the energy is absorbed by the skin and other superficial tissues, resulting in negligible temperature rise in the brain or any other organs of the body.” [10]. The combined, consecutive or simultaneous exposure to RF and solar (UV) radiation of the human skin can easily happen during the everyday life of a human being. Since the RF exposure is classified as a possible human carcinogen (Class. 2B), and the ultraviolet (UV) and solar exposure as a human carcinogen (Class. 1) by IARC, respectively, therefore the combination and consecutive exposure of these two physical agents might have an important role among the environmental stressors. 

### 1.2. Skin Exposure to RF

Skin is the body’s first line of defense. It protects the body against external aggressors like bacteria and fungi, and physical agents like radiations (UV, EMF, temperature). It also has an important role in the immune system of the human body. The skin—because of its generally high exposure—is a target area of interest for RF exposure and other environmental agents. It is affected by several environmental factors such as exposure to chemicals, heat, UV radiation (solar and artificial), etc. The RF exposure from wireless devices represents an additional consecutive exposure to the above-mentioned environmental agents. Nevertheless, the RF exposure is non-ionizing and therefore is considered to be directly non-genotoxic per se [11,12,13]. It is also important to note that mobile wireless systems produce additional conductive thermal exposure on the skin along with RF absorption [14,15,16]. For example, increase of skin temperature due to 20 min exposure to RF of mobile phones was reported by Ghosn et al. [17]. Studies on the effect of RF–EMF on the skin are very scarce [18,19]. Recent data have reported that 20 min of exposure to GSM 900 MHz induces enhanced vasodilatation of skin microvessels during radiofrequency exposure. These effects of radiofrequency exposure on the skin blood flow (SkBF) were seen both during and after acute exposure [20]. Another study also showed that facial SkBF could be modified by mobile phone exposure when the phone was in contact with the skin [21]. The thermal pain of the skin showed a decrease of overall pain ratings of subjective pain perception (VAS), following Universal Mobile Telecommunications System (UMTS) like RF exposure [22].

Regarding the studies of genotoxicity, human skin fibroblasts were intermittently exposed to GSM 1800 MHz RF–EMF with a specific absorption rate of 3.0 W/kg for 1 h or 24 h, followed by immunostaining with anti-gamma-H2AX antibody. The results showed that exposure to RF–EMF for 24 h, significantly induced gamma-H2AX foci formation in human skin fibroblasts (HSFs). However, RF–EMF-elevated gamma-H2AX foci formation in HSF cells did not result in detectable DNA fragmentation, sustainable cell cycle arrest, cell proliferation, or viability change. RF–EMF exposure slightly but not significantly increased the cellular ROS level [23]. Another study suggested that mobile phone radiation might alter protein expression in human skin [24]. Although scarce, these data show that RF–EMF might have a potential effect on the skin. Beyond the frequency range used by current mobile technology in millimeter microwave frequency range, the modifications of gene expressions of a human keratinocyte model was analyzed after being exposed to 60.4 GHz millimeter wave (MMW). No effect of the MMW treatment alone was observed on the human primary keratinocyte transcriptome in non-thermal conditions, and MMW did not alter the ATP contents of the exposed cells [25]. The perception of effects on the skin frequently appears in subjects with idiopathic environmental intolerance, which is attributed to electromagnetic fields (IEI–EMF) and might also have a role in their non-specific symptoms [10,26]. The mechanism by which the body becomes hyper-reactive or hyper-sensitized to electromagnetic energy might start with a totally unrelated toxicant insult or multiple insults in the form of foreign exposures. This pathway to illness has been also referred to as TILT (toxicant-induced loss of tolerance) [27,28]. 

### 1.3. Skin Exposure to UV

Solar radiation is the main source of human exposure to ultraviolet (UV) radiation. Around 95% of ultraviolet radiation that reaches the earth’s surface is UVA (320–400 nm). The most of natural UVB (280–320 nm) and the whole of the UVC (180–280 nm) are blocked by stratospheric ozone. Epidemiological studies have established a causal association between exposure to solar radiation and all major types of skin cancer in humans. Exposure to UV radiation results in specific cyclobutane pyrimidine dimers in DNA. This transition has been seen in premalignant solar keratosis and in malignant skin tumors. Based on these data, the UVB and UVA (UV) radiation is classified as a Class I human carcinogen by IARC [29].

The biological effectiveness of UV radiation is highly dependent on the wavelength [30]. The dose of UV radiation is expressed in an effective radiant dose (J/m^2^), which results from the spectral irradiance multiplied by the spectral weighting curve of biological effectiveness and the exposure time, and is integrated by wavelength. For dermatological use, the standard erythemal dose (SED) was introduced. One SED is a unit of erythemally effective UV radiation, equivalent to 100 J/m^2^ of effective radiant doses.

UV radiation is responsible for a wide variety of different acute and chronic effects on the skin. Acute responses of human skin to UV radiation include photodamage, erythema, mutation, immunosuppression, synthesis of vitamin D, and tanning. Chronic UV radiation effects include photoaging and photocarcinogenesis, which are considered to be induced by mutation and immunosuppression [31]. One of the most important acute effects of UV radiation is DNA photodamage. UVA and UVB show different properties regarding their biological effects on the skin. UVB (280–320 nm) and UVA (320–400 nm) exposure from the sun and from artificial sources (sun beds) are the most important etiological factors for the development of skin cancer, as has been shown by experimental and epidemiological evidence gathered in the past ten years [32]. 

UVB radiation is more cytotoxic and mutagenic than UVA and, according to wavelength dependent studies, is 3–4 orders of magnitude more effective per unit physical dose (J/cm^2^) than UVA for DNA photodamage [33]. UVA, which in contrast to UVB is not filtered by window glass, is able to penetrate deeper into the skin and reach the dermis. UVB can damage DNA directly, in addition, both UVB and UVA can cause indirect DNA damage via the generation of ROS. While UVB is absorbed directly by DNA and induces base structural DNA damage, UVA is mainly responsible for indirect DNA damage by the generation of reactive oxygen species (ROS) that include superoxide anion, hydrogen peroxide, and singlet oxygen, and result in single-strand breaks in DNA and in DNA-protein crosslinks [34]. It was reported that UVA induce double-strand breaks (DSBs) by several authors [35,36]. It is accepted that replication-dependent DSB formation exists [37,38] and replication-independent DSB formation was also shown by several studies [39,40]. ROS are intermediates of the DNA damaging process and thus for the DSB formation. Antioxidants can also reduce DSBs and single-strand breaks (SSBs) [39].

### 1.4. Combined Exposure to RF and Other Physical Agents

There are several research performed in the last decades related to the combined exposure to RF and other physical agents, such as ionizing and UV radiation, or EM exposure without an RF frequency range. These previous works have various working hypothesis that combined (or consecutive) exposure to RF and other radiation might enhance or mitigate the biological effects of exposure to known toxicological agents [41,42,43]. The main hypotheses of these investigations are based on the conclusions of majority of previous studies that the exposure to RF alone is non-toxic or non-genotoxic, below the internationally accepted exposure limits. Another hypothesis of the combined exposure is where the biological effects of exposure to microwaves are mitigated or inhibited by superposition of extremely low frequency (ELF) electromagnetic noise [44,45]. Recently, a specific hypothesis called adaptive response (AR) also appeared in the bioelectromagnetic research where, under a consecutive exposure protocol, the effects of high-dose exposure might be prevented by previous exposure to low doses [46]. This phenomenon is well-studied in the research area of ionizing radiation (IR) and was extended to non-ionizing radiation where the low (adaptive) dose of ionizing radiation was substituted with RF exposure [47]. There are also previous studies on the effects of combined exposure to EM fields and UV radiation, on different mammalian cells or skin [41,48].

The main objectives of the present studies regarding the previously mentioned research were to investigate whether exposure to RF following UV irradiation might enhance the inflammation of the skin due to UV exposure (the protocol called as “Protocol#1”). The secondary aim was to investigate the possible protective effects of RF against UV radiation. Under this second protocol (Protocol#2), the phenomenon of adaptive response was investigated, where pre-exposure of skin to RF (as an adaptive dose; AD) and subsequent exposure to UV radiation (as a challenge dose, CD) was studied. In both protocol, the inflammation of the skin exposed to RF combined with UV radiation was examined on reconstructed 3D human skin model, in vitro, measuring the cytokines (IL-1α, IL-6, and IL-8) and MMP-1 enzyme secretion. In connection with these, our first hypothesis was to check whether the combined exposure of skin to RF radiation might enhance the adverse effect of exposure to UV radiation (Protocol#1), while the second hypothesis was that pre-exposure to RF could prevent the skin from adverse effects of UV radiation (Protocol#2). The adaptive response induced by non-ionizing RF radiation represents a recent new concept in EMF research connected to genotoxicity [47]. We suppose that the combined (i.e., enhanced or protective) effects of exposure to UV and RF—if it exists at all —depend on the sequence and exposure conditions of these two physical agents.

## 2. Materials and Methods

### 2.1. The 3D Full Thickness In Vitro Skin Model

The full thickness (FT) 3D in vitro skin system consists of normal, human-derived epidermal keratinocytes (NHEK) and normal, human-derived dermal fibroblasts (NHDF), which have been cultured to form a multilayered, highly differentiated model of the human dermis and epidermis. Ultrastructurally, it consists of organized basal, spinous, granular, and cornified epidermal layers, analogous to those found in vivo. The dermal compartment is composed of a collagen matrix containing viable normal human dermal fibroblasts. A well-developed basement membrane is present at the dermal/epidermal junction. In the present study, MatTek’s EpiDerm Full Thickness (EFT-300, MaTek Corp, Ashland, MA, USA) reconstructed human skin tissues were used to test inflammation and photoaging (e.g., determination of interleukins and matrix metalloproteinases). The EFT-300 full-thickness in vitro hydrocortisone-free reconstructed human skin (RHS) tissues and hydrocortisone-free maintenance medium (EFT-300-ASY-HCF) were also purchased from MatTek Corporation and were handled according to the manufacturer’s standard user protocols. EFT-300 was supplied as single well tissue culture plate inserts with each insert containing functionally and metabolically active reconstituted skin (with surface area 2.54 cm^2^), shipped at 4 °C, on a medium-supplemented agarose gel. After the shipment, the skin tissues set on inserts were transferred into 6-well plates containing 1-mL medium, equilibrated at 37 °C, 5% CO_2_ overnight, and were maintained in a culture until the start of the experiments. Two tissues were prepared in parallel for each test condition. Throughout the experiments, EpiDermFT was maintained in 35 mm Petri dishes at the air–liquid interface with the lower dermal side of the tissue being exposed to media and the upper epidermal stratum corneum being exposed to air.

### 2.2. Cell Viability–MTT Assay

Inserts with the living tissues were transferred to the cell viability assay, immediately after the experimental protocol (exposures followed by 24 h incubation). MTT assay is a colorimetric assay for assessing cell metabolic activity. Cell viability in the FT tissue models was measured by the enzymatic conversion of the vital yellow dye MTT [3-(4,5-Dimethylthiazol-2-yl)-2,5-diphenyltetrazolium bromide], into a purple formazan salt that was quantitatively measured after extraction from tissues. The MTT assay was carried out with commercially available kits, according to the instructions (MTT Kit–MTT-300, MatTek). In brief, at the end of the protocol EFT-300 tissue samples were placed in a 24-well plate containing 300 µL/well of MTT solution. After 3 h of incubation at 37 °C, 5% CO_2_, each insert was removed carefully, the bottom of the insert from outside was blot-dried on a paper towel, transferred into a new 24-well plate and immersed in a 2 mL/well extraction solution (isopropanol). The plate was covered to reduce evaporation and was incubated for 2 h at room temperature, in the dark, on a shaker. After the extraction process, inserts with the tissues were discarded. The extraction solutions in each well were suspended before transferring 200 µL/sample into a 96-well plate. The optical densities (OD) of the samples were measured at 570 nm. Background readings for all samples were determined at 650 nm and were subtracted to obtain the correct OD. The percentage of viability MTT test was determined for each tissue, using the equation Viability = 100 × [OD(sample)/OD(negative control)]. The percentage of viable cells above 50% was considered according to the tissue manufacturer’s instructions, which followed the OECD 439 Guidelines [49,50].

### 2.3. Matrix Metalloproteinase-1 (MMP-1)

The UV radiation is the primary cause of many adverse biological effects, including photoaging and skin cancer. UV radiation causes DNA damage, protein oxidation, and induces matrix metalloproteinases (MMPs) [51]. UV-induced matrix metalloproteinase-1 (MMP-1) is a crucial biomarker of photoaging [52,53,54]. Certain cytokines, interleukin IL-1α and IL-6 or cell components from UVB-irradiated keratinocytes can regulate MMP-1 expression in fibroblasts, through paracrine effects. Exposure of human skin to UVB or UVA, results in increased MMP-1 production. In our protocol, 24-h incubation time is necessary for the MMP-1 and interleukin concentrations to reach the highest values in the exposed samples [53]. The culture medium from the wells were collected and stored at −20 °C, until measurements. MMP-1 concentration was determined in the culture medium by ELISA.

### 2.4. Cytokines

Inflammation is a physiological response of the body against various insults, such as physical injury, pathogens, exposure to toxic chemicals, and UV irradiation. UV radiation might trigger cutaneous inflammatory responses by directly inducing epidermal keratinocytes to elaborate specific cytokines, such as interleukin (IL-1) and IL-6 [55]. The viable and more basally located keratinocytes respond by releasing interleukin-1 alpha (IL-1α), initiating an acute inflammatory reaction. The proinflammatory cytokine IL-1α is considered a key inducer of the skin inflammatory cascade. After a short period of time, the IL-1α diffuses to the adjacent dermis, and fibroblasts secrete secondary cytokines, such as IL-6 and IL-8. In the end of the exposures, after the 24-h incubation, the culture medium from the wells were collected and stored at −20 °C, until cytokine measurements. These cytokines (IL-1α, IL-6, and IL-8) were measured in the culture medium by ELISA.

### 2.5. Exposure System and Dosimetry

#### 2.5.1. Exposure to Solar UV Radiation

**Figure 1:**
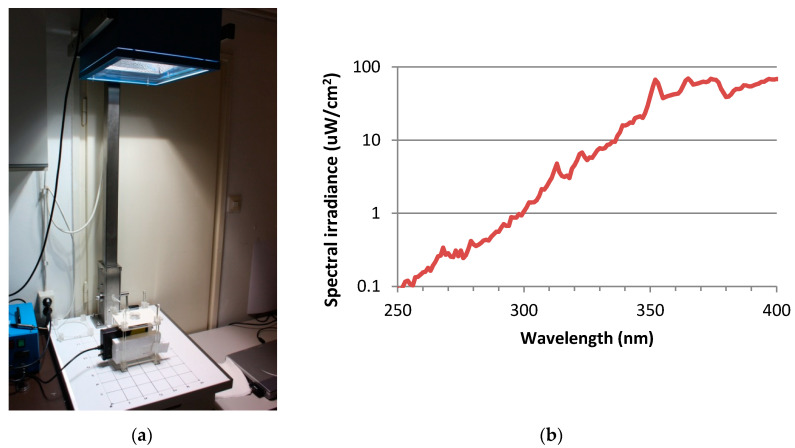
(**a**) Spectral measurement the UV exposure system of the solar simulator (Hönle SOL-500) equipped with the wavelength filter. (**b**) Spectral irradiance of solar simulator filtered by the lid of the petridish.

For UV exposure, the skin tissues were transferred to 35 mm petridishes, filled with 1 mL Dulbecco’s phosphate buffered saline (DPBS) and exposed to doses of 2 SED or 4 SED. For irradiation, a solar simulator SOL-500 (Hönle UV Technology, Gräfelfing, Germany) was used. It was equipped with an H2 filter that transmits wavelength ≥ 295 nm (UVB, UVA, visible light). The distance of the lamp from the tissues was 49 cm, the duration of the exposure was 30 and 60 min for 2 and 4 SED, respectively (Figure 1a). Before exposures, the dose was checked by a calibrated spectroradiometer (ILT-900, International Light, Peabody, MA, USA). As the plastic materials of a cell culture holder contain UV stabilizers, the transmitted spectrum was measured through the same type of 35-mm petridish lid that was used in the assay (Figure 1b). According to the results of the preliminary experiments on the viability of the skin tissues and cytokine production in the Protocol#1, the applied UV dose was 2 SED, while in Protocol#2 the UV dose was set to 4 SED. During the UV exposures, the samples in the petridishes were placed on a double-layered metal box that was connected to the thermal bath with tubes, so the temperature-controlled water could circulate. The temperature of the water in the bath was set to keep the constant temperature below 37 °C, in the samples. The temperature of the DPBS in the petridishes was monitored through the whole duration of the UV exposure and it was kept between 35.5–37.0 °C.

#### 2.5.2. RF Exposure System and Dosimetry

**Figure 2:**
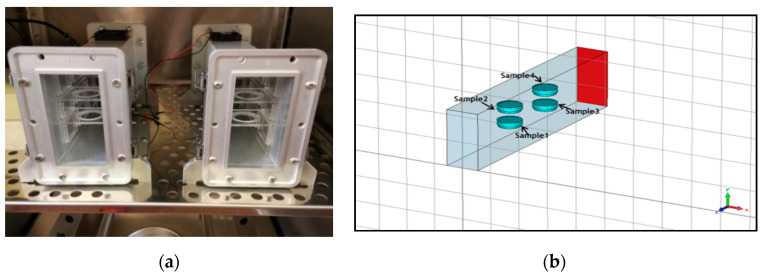
(**a**) Placement of the petridishes in the radiofrequency (RF) waveguide system, and (**b**) modeling for simulation of dosimetry.

**Figure 3:**
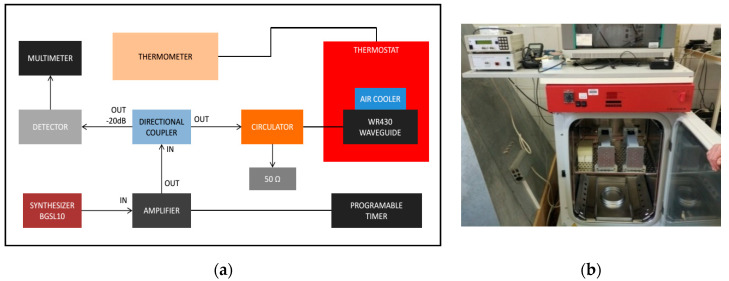
(**a**) Block diagram of the radiofrequency (RF) exposure system and (**b**) the photo of the waveguides placed into the CO_2_ thermostat.

A short-circuited WR-430 resonant waveguide was chosen as the RF exposure chamber at 1.95 GHz. The short-circuited waveguide exposure system was adopted from previous works to optimize efficiency and SAR uniformity inside the in vitro biological samples [56,57]. For the time of RF exposures in the waveguide system, the 3D skin cultures were kept in petridishes on inserts made of Perspex material. The sample aspect was parallel to the electric field vector in the empty waveguide. The distance of the center of the four samples from the short circuit and the relative vertical distance between samples were changed to optimize exposure conditions, according to the maximum of the standing RF waves in the waveguide chamber (Figure 2a,b). Simulations were performed using the commercial software (CST MicrowaveStudio^®^, Computer Simulation Technology, Darmstadt, Germany, 2016), based on the Finite Integration Technique, to evaluate scattering parameters, as well as SAR distribution inside the samples. The standard deviation over mean value was considered as a measure of the SAR non-homogeneity inside the sample. Both sham and exposed short-circuited waveguide systems were placed in a CO_2_ thermostat and air ventilation was also provided (Figure 3a,b). The measurement of the temperature within the sample was performed by four channels of non-perturbing optical temperature probes (LumaSense Technologies Inc., Santa Clara, CA, USA) and the temperature elevation was kept below 1 degree of Celsius. The 3G UMTS (Universal Mobile Telecommunications System) systems used WCDMA (Wideband Code Division Multiple Access) modulation, which is a non-periodic cocktail of signals with a 5 MHz bandwidth. The WCDMA signal cocktail was generated by a specific UMTS synthesizer (Bonn-Hungary Ltd., Hungary), according to the requirements of RF bioelectromagnetic studies [58]. Since the output RF power level of the UMTS synthesizer is limited, an RF power amplifier (Bonn-Hungary Ltd., Hungary) was connected to guarantee the necessary RF power to provide the SAR up to 4 W/kg in the samples.

The level of SAR of the exposed 3D tissues depended on the study performed by combined exposure under Protocol#1 or Protocol#2. In case of Protocol#1, following 2 SED UV irradiation, the samples were exposed to RF with 20 min switched on/off time frames, during a 24-h exposure period, where the SAR level was 4 W/kg. Under Protocol#2, where the possible adaptive response was investigated, a 24 h of continuous exposure to RF with 1.5 W/kg of SAR (as adaptive dose) was used according to the preliminary studies published by other research teams [47] and the UV radiation (as challenge dose) was 4 SED, respectively.

### 2.6. Experimental Protocols

**Figure 4:**
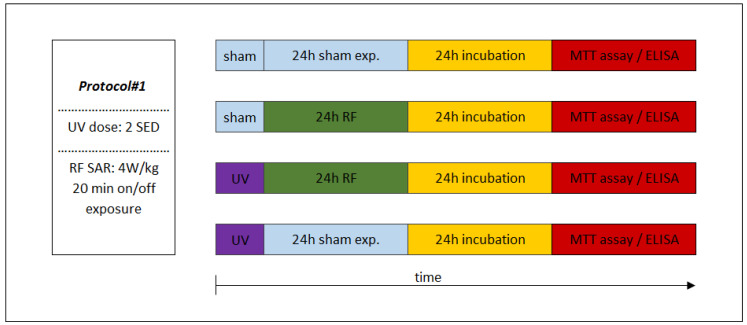
Experimental protocol of combined consecutive exposure for investigation of cooperative effects of exposure to RF and UV. Under Protocol#1, the skin tissues were first exposed to 2 standard erythemal dose (SED) UV and then 24 h 1950 MHz WCDMA (UMTS) modulated RF for 20 min on/off exposure with 4 W/kg SAR.

**Figure 5:**
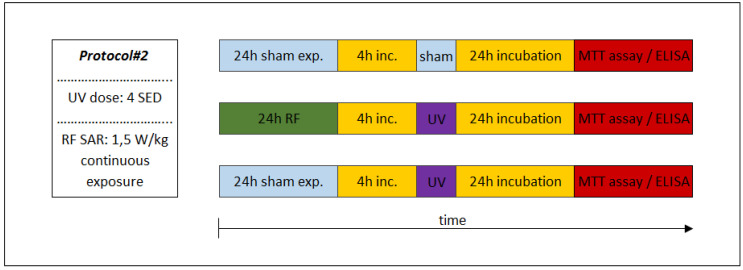
Combined consecutive exposure for the investigation of possible adaptive response. Under Protocol#2, the cells were first pre-exposed to 1950 MHz WCDMA (UMTS)-modulated RF (24 h, 1.5 W/kg) as an adaptive dose and then after 4 h, the incubation followed the UV radiation of 4 SED as a challenge dose.

The sequence of exposures to UV and RF depended on the appropriate study protocol. In the case of combined exposure of Protocol#1, the UV 2 SED irradiation (as first exposure) was followed immediately by 24-h RF irradiation with 4 W/kg, as a second exposure (Figure 4). Under Protocol#2, the cells were pre-exposed to RF (24 h, 1.5 W/kg) and after 4 h of incubation the UV radiation was set to 4 SED (Figure 5). Different UV doses were chosen because in case of possible cooperative effect (Protocol#1), a lower UV dose (2 SED) is needed to detect if there might be additional effects from RF exposure, while in the case of an adaptive response, the protective effect is more detectable if the effect of exposure to UV is stronger due to a higher (as challenge 4 SED) UV dose.

Under Protocol#1, the biological assays of inflammation and photoaging was tested after exposure to UV (2 SED in 30 min), followed by RF intermittent exposure (4 W/kg, 24 h), in order to investigate presumable cooperative effects of these two physical agents. In this procedure, 24 h incubation was necessary after UV and RF exposure protocols, when the secretions of the MMP-1 and interleukin concentrations could reach the highest values [53]. The sham-exposed group served as a negative control, where the samples were kept in the same conditions as that of the exposed samples, but in the absence of any radiation.

The experimental Protocol#2 is shown in Figure 5, where the possible protective phenomenon (i.e., adaptive response) was investigated. In this exposure protocol, the adaptive dose (AD) was 24 h of RF exposure and after this, the challenge dose was 4 SED UV radiation expressed in 1 h. The time interval between the adaptive and challenge doses was 4 h. Two other groups of samples were exposed to UV alone or to the sham, as a negative control. In order to evaluate the secretion of MMP-1 and interleukins before the test, 24 h of incubation was necessary, as discussed above.

In the case of both protocols, following the combined exposures of UV and RF, the tissues were incubated at 37 °C and 5% CO_2_ for 24 h and the cell culture medium was collected and frozen at −20 °C, until analysis. The concentration of IL-1α, IL-6, and IL-8 and MMP-1 was measured in the collected culture media with ELISA Kits (Thermo Fisher Scientific, USA). Viability was evaluated 24 h after exposures with MTT test on the full thickness skin tissues, as described in 2.2 [59,60].

### 2.7. Statistical Analysis

Two biological parallels were used for each type of treatment in all three independent experiments. For the MTT assay, the mean percentage of the viability was calculated for each treatment. Data from the three independent experiments were statistically analyzed by ANOVA. For the interleukins and MMP-1 analyses, two parallels were used for a sample in ELISA measurements. Data of the interleukins and the MMP-1 concentrations were analyzed by the linear mixed effect models with Tukey’ post-hoc test. The significance level was set at *p* < 0.05. All analyses were carried out using R Studio version 1.1.463 software (Software Foundation Inc., Boston, MA, USA). 

## 3. Results

### 3.1. MTT Assay

The MTT Assay (MTT-300) determined the viability of the reconstructed 3D tissues. If the percentage viability value of the treated samples had dropped to 50%, compared to the sham-exposed (non-treated) samples, it was considered non-viable. In our experiments, one tissue did not reach this value, therefore, we excluded it from the evaluation. The viability values of all other samples were above 50% (61–100%). Table 1 shows the results of viability expressed in percentage of sham-exposed tissues. In Protocol#2, the viability decreased significantly, following the treatment of both RF + UV and UV alone, compared to the sham-exposed samples.

### 3.2. Cooperative Effects of RF (Protocol#1)

**Figure 6:**
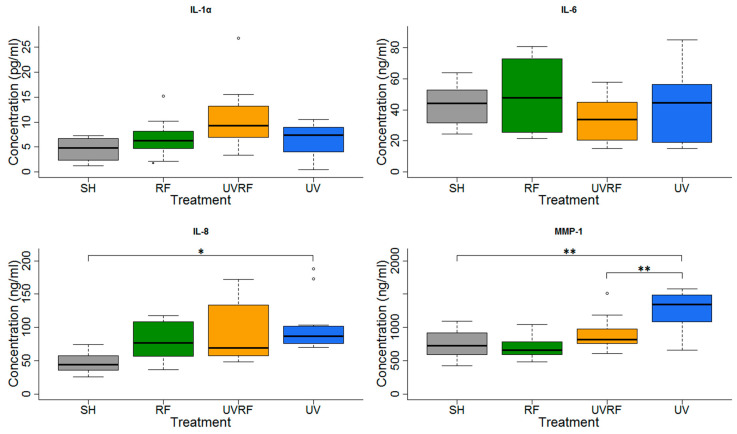
Results of the experiments performed under Protocol#1 where the skin tissues were first exposed to 2 SED UV and then 24 h 1950 MHz WCDMA (UMTS), modulated RF 20 min on/off exposure with 4 W/kg SAR. Data represent three independent experiments; * *p* < 0.05, ** *p* < 0.01.

In the experiments performed under Protocol#1, the skin tissues were first exposed to 2 SED UV and then to 4 W/kg RF (Figure 6). In the IL-1α cytokine concentration, there were no significant changes between the treatments, but slight increase in the UV + RF treatment could be observed. No significant changes in IL-6 concentration were found. There was a significant increase in the IL-8 concentration in only the UV-treated samples, as compared to the sham-exposed samples. The IL-8 concentration of the tissues exposed to RF alone was not significantly different from the sham-exposed tissues. Moreover in the UV + RF treatment, the RF exposure slightly (but not significantly) decreased the IL-8 concentration caused by UV. The concentration of the MMP-1 enzyme in the samples treated by UV alone was significantly higher than that in the samples treated by sham exposure or UV + RF. The samples treated only by RF was not significantly different from any other samples.

### 3.3. Protective Effects (Adaptive Response) (Protocol#2)

**Figure 7:**
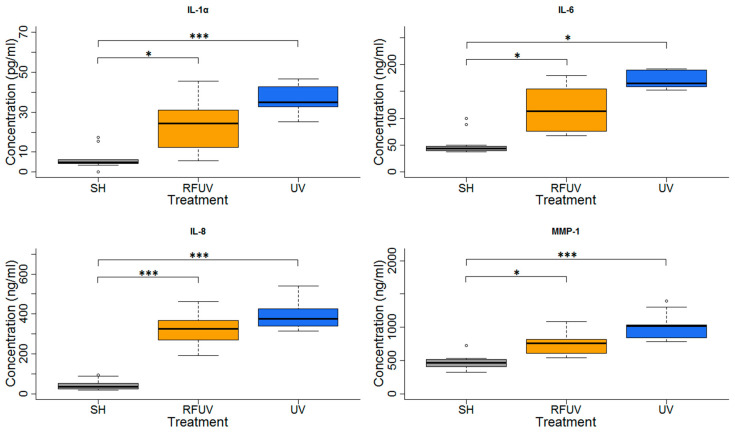
Results of the experiments performed under Protocol#2 where pre-exposure of tissues with 1950 MHz WCDMA (UMTS) modulated RF 1.5 W/kg for 24 h and subsequently with 4 SED UV radiation. Data represent three independent experiments; * *p* < 0.05, *** *p* < 0.001.

In the experiments performed under Protocol#2, the skin tissues were first exposed to 1.5 W/kg RF and then to 4 SED UV. In the case of these experiments, all type of interleukins as well as MMP-1 concentration increases were statistically significant, following exposure to 4 SED UV. The elevation of interleukins (IL-1α, IL-6, and IL-8) was higher compared to the 2 SED UV irradiation performed under Protocol#1. The change of the concentration in all interleukins and MMP-1 was not significant when the tissues were treated with pre-exposure to RF with 1.5 W/kg SAR for 24 h, and subsequently with 4 SED UV radiations. Nevertheless, a slight tendency of the protective effects, namely adaptive response, were observed in all interleukins and also in MMP-1 production (Figure 7).

## 4. Discussion

Radiofrequency electromagnetic radiation belongs to the non-ionizing part of the electromagnetic spectrum, as all UV light greater than 100 nm wavelength are too weak to ionize molecules. Permissible RF levels proposed by ICNIRP (1998, 2020) are based on thermal effects only, whereas, a large number of publications suggest non-thermal effects, mainly through free radicals and oxidative stress [61,62,63,64,65,66]. Our work is based on the assumption of cooperative (additive), synergistic, or possible protective mechanisms of successive exposure to RF and UV, since UV radiation is able to induce adverse biological effects, per se. In our everyday life, the combined exposure of skin to UV and RF is considerable and happens frequently. The subjects were exposed consecutively to UV and RF using their mobile phones. Since we intended to mimic the RF exposure from mobile phones rather than base stations we decided to use intermittent RF exposure, similarly to several studies performed previously [67,68,69]. The higher frequency of RF radiation towards millimeter waves of microwaves, produced higher absorption in the skin and the superficial tissues of the body. The investigation on the role of UV radiation and combined exposure with RF within these inflammation processes might add important knowledge about the combined effects of solar UV and RF radiation. The investigation of the combined effects of UV and RF exposure on the skin become more and more important, particularly before the deployment of 5G mobile technology. 

The results of our present studies suggest that RF exposure alone does not induce inflammation process in 3D skin in vitro model, as was found by others [70]. Similarly, RF did not significantly enhance the inflammation process induced by UV radiation, although RF exposure slightly enhanced the IL-1α induced by UV, under our experimental conditions. The IL-1 family of cytokines comprises proteins with pro- and anti-inflammatory functions. The proinflammatory signaling is also activated by IL-1 family members such IL-1α [71]. Dysregulation of the IL-1 system might lead to diseases such as psoriasis, atopic dermatitis, contact dermatitis, and cutaneous lupus erythematosus. These inflammatory skin conditions greatly affect quality of life and life expectancy, and their frequencies are increasing. 

It was also concluded that there is a slight non-significant adaptive (protective) response of RF exposure on the effect of inflammation in the skin caused by UV radiation generated by a solar simulator lamp. Originally the adaptive response describes the phenomenon where a relatively low dose of ionizing radiation as an adaptive dose, induces a kind of adaptation (sometimes referred to as resistance) to genetic damage induced by a subsequently higher dose of IR (challenge dose, CD) [46]. A possible hypothesis for this response is that exposure to RF at low levels might activate the alarm signals at a much earlier stage than under normal function. This activation might result in better protection against, e.g., subsequent exposure to a toxic agent, in our case, against UV solar radiation. AR is not only inducible via the same agent, but there is a kind of “cross-resistance” to similar genotoxic agents [47]. Combined exposures to different agents such as non-ionizing radiation (as AD) and chemicals/pharmaceuticals or IR (as CD) also induce the AR phenomenon [72]. For example, the AR was studied in human lymphocytes in vitro after exposure to RF at 1950 MHz as AD, and later to 1.0 or 1.5 Gy as CD [73]. It was shown that the IR induced a significant decrease in the micronuclei incidence rate when the lymphocytes were pre-exposed to RF–EMF. In earlier studies of Sannino and colleagues [74,75], AR was also detected in human lymphocytes using 900 MHz RF–EMF and the chemical mutagen mitomycin C.

Our results are promising in the sense that even if the effect was not significant, the trend towards a protective effect was visible. One could argue that the duration of the exposure (long-term, for example) or the intensity of the RF applied or the combination of both could be an important factor likely to influence the significance of this trend or the opposite. With regard to the electrohypersensitive individuals who reported several symptoms related to the skin, such as itching, redness, burning sensation, as a consequence of RF exposure, a recent study on the effect of RF effect on the electrodermal activity (EDA) was performed. This did not show any changes in this physiological parameter [76]. Our present study, in line with other studies [77,78], did not support the hypothesis of a direct cause-effect relationship between RF exposure and dysfunction of the skin for certain parameters.

Another important fact, the solar radiation covers a broad range of wavelengths of photon energy including ionizing radiation, ultraviolet radiation, visible light, and infrared radiation. Solar ultraviolet radiation has several adverse effects, such as sunburn, development of solar lentigines, and triggering inflammation and immunosuppression, which mediate tumor growth. Inflammation caused by sunburn promotes carcinogenesis and in particular DNA lesions are implicated. The sunburn process is dependent on several factors, including UV dose, UV wavelength, and photoskin type. After the cellular molecules absorb the UV radiation, photochemical reactions occur, and these processes are responsible for biological changes that culminate in sunburn. It was also reported that large doses of UV radiation, which cause irreparable damage to cells, induce DNA double-strand breaks and increase secretion of IL-6 [79]. It was also suggested that the UV response is initiated at or near the cell membrane rather than in the nucleus, and that the response might be elicited by oxidative stress caused by UV radiation. UV radiation triggers sequential molecular responses, thereby activating cell-surface growth factors and pro-inflammatory cytokine receptors. In those studies in which fibroblast–keratinocyte cell interactions (paracrine signaling) are important, a full thickness (FT) in vitro skin model was used. Using the 3D skin tissue model is important in the context of the present study to evaluate the cytokine production. The MatTek’s EpiDermFT 3D tissue model, which was used in our study, consisted of normal, human epidermal keratinocytes (NHEK) and normal, human dermal fibroblasts (NHFB), cultured to form a multilayered model of the human epidermis and dermis [80,81].

The environmental RF exposure covers a wide range of electromagnetic radiation. The most important frequency bands within the RF radiation derive from the mobile systems, including handset and base stations. The environmental RF exposure levels are usually below the regulatory limits of the International Commission of Non-Ionizing Radiation Protection (ICNIRP), and is considered to be a non-genotoxic agent. However, due to the increasing UV radiation worldwide, skin exposure to UV has become more and more important. Since the absorption of RF from mobile phones by the human skin tissue is considerable, our investigation of combined exposure to RF and UV is important from the aspects of radiation biology and bioelectromagnetics.

## 5. Conclusions

In the present study, we investigated the combined exposure of RF and UV, since the human skin is exposed to both radiation in our normal life. We found that RF exposure generated by 3G mobile systems at 1950 MHz under the described exposure conditions, did not induce an inflammation process in the skin tissue in vitro. We found that a 24-h RF exposure significantly reduced the MMP-1 enzyme concentration, caused by prior UV exposure. Another important finding of our study is that RF exposure did not significantly enhance the inflammation process induced by UV radiation. On the other hand, we concluded that there was a slight non-significant adaptive (protective) response of inflammation in the skin caused by UV radiation when the skin was pre-exposed to RF. The investigation of the possible adverse effects on the skin due to the high frequency electromagnetic fields become more and more important before the deployment of 5G mobile systems. Using this new technology, the absorption of exposure to RF in the skin will be enhanced. The skin will be the most important target organ of the RF exposure to 5G. Therefore, our approach of combined (i.e., consecutive) exposure to UV and RF might be important in future research related to 5G and skin.

## Figures and Tables

**Table 1:** The results of the MTT assay of the treatments by Protocol#1 and Protocol#2. Data represent the mean and the standard deviation (SD) of the three independent experiments normalized to the sham-exposed tissues (100%).

**Protocol#1**		
**Treatment**	**% Viability**	
	**Mean ± SD**	***p*-Values ^1^**
SH	100 ± 2.7	
RF	99.15 ± 3.17	0.996
2 SED UV + RF	91.95 ± 3.89	0.245
2 SED UV	89.54 ± 8.16	0.105
**Protocol#2**		
**Treatment**	**% Viability**	
	**Mean ± SD**	***p*-Values ^1^**
SH	100 ± 9.21	
RF + 4 SED UV	83.34 ± 9.35	0.052
4 SED UV	79.23 ± 6.39	0.019

^1^*p*-values are based on the comparison to the sham-exposed treatment.
